# Antitumor Responses of Invariant Natural Killer T Cells

**DOI:** 10.1155/2015/652875

**Published:** 2015-10-12

**Authors:** Jennie B. Altman, Adriana D. Benavides, Rupali Das, Hamid Bassiri

**Affiliations:** ^1^Division of Infectious Diseases, Children's Hospital of Philadelphia, Philadelphia, PA 19104, USA; ^2^Division of Oncology Research, Children's Hospital of Philadelphia, Philadelphia, PA 19104, USA

## Abstract

Natural killer T (NKT) cells are innate-like lymphocytes that were first described in the late 1980s. Since their initial description, numerous studies have collectively shed light on their development and effector function. These studies have highlighted the unique requirements for the activation of these lymphocytes and the functional responses that distinguish these cells from other effector lymphocyte populations such as conventional T cells and NK cells. This body of literature suggests that NKT cells play diverse nonredundant roles in a number of disease processes, including the initiation and propagation of airway hyperreactivity, protection against a variety of pathogens, development of autoimmunity, and mediation of allograft responses. In this review, however, we focus on the role of a specific lineage of NKT cells in antitumor immunity. Specifically, we describe the development of invariant NKT (iNKT) cells and the factors that are critical for their acquisition of effector function. Next, we delineate the mechanisms by which iNKT cells influence and modulate the activity of other immune cells to directly or indirectly affect tumor growth. Finally, we review the successes and failures of clinical trials employing iNKT cell-based immunotherapies and explore the future prospects for the use of such strategies.

## 1. Introduction

Natural killer T (NKT) cells are innate-like lymphocytes typified by coexpression of receptors characteristic of natural killer and conventional T cells [1]. As such, murine NKT cells generally bear Ly49 receptors, NKG2 family of receptors, CD94, and NK1.1 (the latter only being expressed in specific strains, including the commonly used C57BL/6). Human NKT cells often express similar surface molecules including CD56, CD161, CD94, NKG2D, and NKG2A. Both human and mouse NKT cells display a variety of stimulatory and inhibitory T cell-associated receptors and ligands (e.g., CD28 and CD154), whose expression depends on the activation status of the cell. Finally, both human and murine NKT populations include CD4^+^ and CD4^−^CD8^−^ (double negative; DN) subpopulations; while CD8^+^ NKT cells are found in humans, they are rare in mice [2].

The T cell receptors (TCRs) expressed by NKT cells recognize the conserved and nonpolymorphic MHC class I-like molecule, CD1d. Unlike classical MHC class I-like molecules, the expression of CD1d is largely restricted to cells of bone marrow origin including antigen presenting cells (APCs) such as dendritic cells (DCs), macrophages, and B cells. Furthermore, the CD1d molecule (via heterodimerization with *β*_2_-microglobulin) specializes in displaying lipid moieties rather than protein polypeptides. Importantly, intact expression of CD1d is critical for the development of NKT cell populations, as* Cd1d−/−* mice are devoid of these cells [3]. NKT cells are further subclassified into Type I or II lineages, depending on the composition of their TCR and the CD1d-presented glycolipid antigens to which they respond. Type I or invariant NKT (iNKT) cells express canonical TCR*α* chains comprised of specific gene segments (V*α*14-J*α*18 in mice and V*α*24-J*α*18 in humans) that preferentially pair with specific TCR*β* chains (V*β*8, V*β*7, or V*β*2 in mice and V*β*11 in humans). These invariant TCR*αβ* pairings confer reactivity to CD1d and a restricted array of presented glycolipid antigens. The dependence of iNKT cells on the V*α*14-J*α*18-comprised TCR*α* is demonstrated by* Vα14* TCR transgenic mice, in which a higher frequency and number of iNKT cells are observed [4], and also* Jα18−/−* mice, in which no mature iNKT cells develop [5]. Despite the conserved use of the invariant TCR, iNKT cell populations are phenotypically (e.g., presence or absence of CD4 expression) and functionally (e.g., preferential production of certain cytokines, such as IL-17) diverse.

The prototypical (and first discovered) iNKT cell stimulatory glycolipid, alpha-galactosylceramide (*α*-GalCer), was identified during a screening for compounds from marine sponges (*Agelas *species) with antitumor activity [6]. Since this initial discovery, a number of naturally occurring and synthetic lipid antigens have been described to bind CD1d and activate iNKT cells. These cells are now typically identified using CD1d tetramers loaded with *α*-GalCer or its synthetic analogs (e.g., PBS-57; [7]). In contrast, Type II or variant NKT (vNKT) cells bear a more diverse array of TCR*α* and *β* chains and have been shown to recognize sulfatide moieties presented by CD1d [8]. More recently, Type II NKT cells have also begun to be better characterized through development of CD1d tetramers loaded with sulfatide [9, 10], but these cells are still less well characterized than their invariant brethren. Given that far more is known regarding the antitumor activity of iNKT cells, we will predominantly focus our attention on these cells.

## 2. iNKT Cell Development and Acquisition of Effector Function

iNKT cells develop in the thymus, by originating from CD4^+^CD8^+^ double positive (DP) thymocytes. Positive selection of iNKT cells is mediated by homotypic interactions of DP cells and recognition of glycolipid antigen-CD1d complexes [11–14]; however, the nature of the self-antigens involved in this process remains somewhat elusive. Like conventional T cells, maturation of iNKT cells at the DP stage and beyond depends on the ability to construct a functional TCR and intact signaling. As such, iNKT cells are profoundly diminished or absent in mice lacking expression of RAG, CD3*ζ*, Lck, ZAP-70, SLP-76, ITK, LAT, or Vav [15–21]. Transcriptionally, development of iNKT cells at the DP stage is regulated by the transcription factor ROR*γ*t, which prolongs the survival of DP thymocytes by upregulating Bcl-X_L_, to allow sufficient time for distal TCR*α* gene segment rearrangements to occur [22, 23]. More recent studies have shown that HEB, the E protein family of basic helix-loop-helix transcription factors, regulates iNKT cell development by regulating ROR*γ*t and Bcl-X_L_ mRNA [24]. Finally, the absence of the transcription factor Runx1 also blocks iNKT cell development at the earliest detectable iNKT cell-committed subset [23].

iNKT cell development at the DP stage also critically depends on the signals generated by engagement of the Signaling Lymphocyte Activation Molecule (SLAM) family of surface receptors, which are expressed on developing iNKT cells, as well as conventional DP thymocytes. SLAM family receptor signaling is transduced by the adaptor molecule SAP (SLAM-associated protein), which in turn binds to the tyrosine kinase Fyn, and results in propagation of a phosphorylation signal [25, 26]. Accordingly, iNKT cells fail to develop in mice and humans bearing mutations in the gene that encodes for SAP [27–29], in mice lacking Fyn or expressing a mutant version of SAP that cannot bind Fyn [23, 30], in mice in which both Ly108 and SLAM signaling are simultaneously disrupted [31], and in those lacking the transcription factor* cmyb* (which is necessary for appropriate expression of SAP and certain SLAM family members) [32]. Taken together, these studies establish the importance of the SLAM-SAP-Fyn signaling axis in iNKT cell development.

Following positive selection, iNKT cells undergo distinct stages of maturation that are characterized by the sequential acquisition of CD24, CD44, and NK1.1: CD24^hi^CD44^lo^NK1.1^−^ (Stage 0), CD24^lo^CD44^lo^NK1.1^−^ (Stage 1), CD24^lo^CD44^hi^NK1.1^−^ (Stage 2), and finally CD24^lo^CD44^hi^NK1.1^+^ (Stage 3) [33]. As these cells progress through these developmental stages, they begin to upregulate NK cell markers (e.g., NKG2D and Ly49 receptors), CD69, and CD122 and acquire distinct effector functions (e.g., production of IL-4, IFN-*γ*, perforin, and granzymes) [34]. Acquisition of these effector functions is tightly regulated by several transcription factors [35]. One of the key regulators of iNKT cell development and acquisition of an effector/memory phenotype and functions is the broad complex tramtrack bric-a-brac-zinc finger transcription factor PLZF, whose expression is highest in Stage 0 and 1 populations [36, 37]. PLZF-deficient animals exhibit a severe reduction in iNKT cell number and PLZF-deficient iNKT cells fail to cosecrete Th1 and Th2 cytokines upon stimulation [36, 37]. Recently, it was demonstrated that the lethal-7 microRNA posttranscriptionally regulate PLZF expression and iNKT cell effector functions [38].

The transcription factor T-bet is indispensable for the final maturation stages of iNKT cells [39, 40] and absence of this transcription factor results in reduced iNKT cell numbers due to developmental blockade at Stage 2. T-bet-deficient iNKT cells fail to proliferate in response to IL-15 as they lack surface expression of CD122, a component of the IL-15 receptor [40]. In addition, T-bet-deficient iNKT cells fail to produce IFN-*γ* in response to TCR stimulation and exhibit defective cytolytic activity [39, 40] as T-bet directly regulates the activation of genes associated with mature iNKT cell functions, such as perforin, CD178, and IFN-*γ* [40].

As iNKT cells progress to Stage 1, a proportion of cells downregulate CD4, giving rise to DN iNKT cells. Generation of the CD4^+^ iNKT cell lineage and production of Th2-type cytokines is critically regulated by the transcription factor GATA-3. Similar to PLZF-deficient iNKT cells, GATA-3 deficient iNKT cells fail to produce Th1 or Th2 cytokines in response to *α*-GalCer [41]. Recent studies have identified a unique subpopulation of NK1.1^−^CD4^−^ iNKT cells that are transcriptionally regulated by ROR*γ*t and capable of producing large quantities of IL-17 upon stimulation [42]. As such, iNKT cells are also sometimes classified into NKT1, NKT2, and NKT17 based on their cytokine production profiles and respective expression of T-bet, GATA-3, and ROR*γ*t [43, 44]. Finally, mechanistic target of rapamycin (mTOR) signaling has also been shown to be important for iNKT cell lineage diversification and acquisition of effector functions [45–48], and loss of mTOR2 may result in loss of NKT17 cells. Taken together, these recent studies provide new insights into the transcriptional regulation of iNKT cell maturation and functional differentiation.

## 3. iNKT Cells and Antitumor Immunity

The importance of iNKT cells in mediating protection against tumors is highlighted by several findings. First, a number of independent studies have shown a decrease in the number of iNKT cells in the peripheral blood of patients with a variety of cancers and even precancerous myelodysplastic syndromes [49–51]. Moreover, the iNKT cells that persist appear to have decreased proliferative and functional responses [52–54]. Interestingly, an increased frequency of peripheral blood iNKT cells in cancer patients portends a more favorable response to therapy [55, 56]. While these observations identify an association between iNKT cell numbers and/or function and development of malignancy, they do not provide a direct causal link. This link has been established in a number of mouse studies in which the biology of the host and initiation of tumors can be more systematically manipulated via gene knockouts, antibody depletion strategies, and adoptive transfer of various lymphocyte populations into cancer-predisposed or tumor-challenged hosts.

In mice that are prone to development of tumors due to loss of one allele of a tumor suppressor (*p53+/−*), absence of iNKT cells (by virtue of genetic knockout of the J*α*18 gene segment or CD1d) results in earlier and more frequent development of tumors and thus shorter survival [57], when compared to iNKT-sufficient littermates. Similarly, treatment of* Cd1d−/−* and* Jα18−/−* mice with a carcinogen resulted in increased incidence and earlier onset of tumors in comparison to treated wild type mice [58]. Conversely, administration of *α*-GalCer to mice controlled the growth and metastasis of adoptively transferred [59, 60] or carcinogen-induced [61, 62] or spontaneous [63] tumors. Moreover, adoptive transfer of iNKT cells into* Jα18−/−* iNKT cell-deficient mice prevented the growth of subcutaneous sarcomas [62]. Finally, adoptive transfer of small numbers of purified iNKT cells into lymphocyte-deficient NOD-*Scid*-*IL2rγ−/−* (NSG) mice was sufficient to protect mice from challenge with a CD1d^+^ tumor [64]. These findings collectively argue that iNKT cells play a central and nonredundant role in the response to tumors. Further studies would shed light on the mechanisms by which iNKT cells exert these antitumor effects.

### 3.1. Indirect Cytokine-Mediated Modulation of Antitumor Responses

Engagement of the invariant TCR by CD1d/glycolipid antigen complexes results in iNKT cell activation, an event that is typified by rapid and robust production of a variety of cytokines and chemokines, including—but not limited to—IL-2, IL-4, IL-10, IL-13 IL-17, IFN-*γ*, TNF*α*, TGF*β*, GM-CSF, RANTES, eotaxin, MIP-1*α*, and MIP-1*β* [65, 66]. The nature and magnitude of the iNKT cell cytokine response depend on the glycolipid antigen; for example, *α*-GalCer-mediated iNKT cell activation elicits a strong IFN-*γ*-dominated cytokine response, while OCH (a synthetic analog of *α*-GalCer with a truncated lipid chain) elicits a response with significantly higher level of IL-4 production [67]. The rapidity of this cytokine response is attributed to the semiactivated state of iNKT cells and the presence of preformed cytosolic mRNA for a variety of cytokines [68]. Indeed, administration of *α*-GalCer to iNKT cell-sufficient, but not iNKT cell-deficient, mice results in polyclonal activation of conventional T, B, and NK cells within 3-4 hours [69] and also eventually leads to the mobilization of macrophages and neutrophils [70]. Intriguingly, it was previously believed that mammalian species are incapable of producing glycolipids (such as *α*-GalCer), in which the sugar moiety is attached via an O-linkage to the ceramide backbone in an alpha-anomeric configuration. Despite the absence of *α*-glucosyl or *α*-galactosyl transferases in mammals, recent findings indicate that a small percentage of the glycolipids that are constitutively presented by mammalian CD1d are indeed *α*-anomeric [71]. Whether the percentage of CD1d-presented *α*-anomeric glycolipids is altered in tumor tissues represents an interesting question that deserves further future investigation.

**Figure 1:**
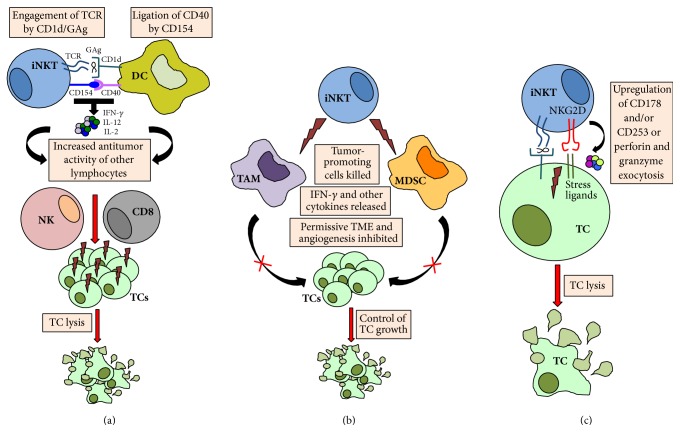
Possible mechanisms of iNKT cell-mediated antitumor responses. (a) Indirect cytotoxicity. iNKT cells and DCs reciprocally coactivate each other via TCR:CD1d/GAg and CD40:CD154 interactions, resulting in the release of several cytokines that secondarily activate and promote the antitumor cytotoxicity of other effector lymphocytes. (b) Modulation of the TME. iNKT cells kill tumor-supporting cells, such as TAMs and MDSCs, and also limit angiogenesis, to indirectly control tumor growth. (c) Direct cytotoxicity. iNKT cells mediate lysis of tumor targets via engagement of TCR or NKG2D. Lightning bolt: exertion of direct cytotoxicity; DC: dendritic cell; GAg: glycolipid antigen; TC: tumor cell; TAM: tumor-associated macrophage; MDSC: myeloid-derived suppressor cell; TME: tumor microenvironment.

Nonetheless, following encounter with CD1d/antigen complexes displayed by APCs, iNKT cells not only produce cytokines but also upregulate surface expression of CD154 (see Figure 1(a)). Ligation of APC-expressed CD40 is especially important for mediating subsequent maturation and functional activation of DCs, subsequent upregulation of CD80 and CD86, and amplified production of IFN-*γ* [72, 73]. In addition, the ligation of the chemokine receptor CXCR6 on iNKT cells by CXCL16 expressed on APCs also provides costimulatory signals resulting in robust *α*-GalCer-induced iNKT cell activation [74]. Importantly, matured DCs are potent producers of IL-12, which induces sustained IFN-*γ* production by iNKT cells [75–77]. The importance of iNKT cells in IL-12-mediated tumor rejection was effectively demonstrated by the defective clearance of a variety of tumors in* Jα18−/−* mice [5]. Mature DCs also support the priming and activation of CD8^+^ T cells, culminating in optimal effector and memory cell formation [72, 78]. Finally, the sustained release of IFN-*γ* by iNKT cells leads to activation and proliferation of NK cells and NK cell secretion of IFN-*γ*. The combination of cytokines (e.g., IL-2, IL-12, and IFN-*γ*) as a result of iNKT cell activation also leads to upregulation of death-inducing ligands (e.g., CD178 or CD253) on NK cells and CD8^+^ T cells [79, 80]. These sequential activation events are believed to be critical for the *α*-GalCer-induced iNKT cell-mediated antitumor effects [76, 81, 82]. As such, iNKT cells not only bridge the activation of innate and adaptive immunity, but also indirectly potentiate the antitumor activity of other cytotoxic effector lymphocytes.

### 3.2. Indirect Control of Tumor Growth via Alteration of Tumor Microenvironment

Tumor establishment and growth are believed to be intricately modulated by a myriad of soluble and contact-derived signals obtained from the tumor microenvironment (TME), which consists of the tumor cells themselves, tumor-infiltrating lymphocytes (TILs), and stromal cells that communicate in a dynamic and bidirectional manner. In addition to their indirect modulation of other effector lymphocyte populations, iNKT cells may also regulate tumor growth via their effects on the TME (see Figure 1(b)). Indeed following intravenous administration, iNKT cells were shown to represent a significant percentage of the TILs in patients with head and neck carcinomas [83, 84]. Importantly, higher frequency of tumor-infiltrating iNKT cells correlated with overall and disease-free survival as an independent prognostic factor in primary colorectal cancer patients [85] and with tumor regression in head and neck carcinomas [86]. Conversely, in patients with primary hepatocellular or metastatic cancer, CD4^+^ iNKT cells that produced high levels of Th2-type cytokines and had low cytolytic activity were enriched within the tumor and appeared to inhibit the expansion of antigen-specific CD8^+^ T cells, suggesting that these particular iNKT cells may contribute to generate an immunosuppressive microenvironment [86].

In experimental studies, cotransfer of human monocytes and iNKT cells to tumor-bearing NOD-*Scid *mice suppressed tumor growth when compared with mice that received monocytes alone [87]. Importantly, iNKT cells can target tumor supportive cells such as tumor-associated macrophages (TAMs), a highly plastic monocyte-derived subset of inflammatory cells that can exert immunosuppressive functions, and promote tumor proliferation and matrix turnover [88, 89]. Indeed TAMs are known to produce IL-6, a cytokine that appears to promote the proliferation of many solid tumors, including neuroblastomas and breast and prostate carcinomas [87]. Consistent with the tumor-permissive capacities of TAMs, Chen et al. found that macrophage density correlated positively with microvessel counts and negatively with patient relapse-free survival [90]. Since TAMs cross-present neuroblastomaderived endogenous CD1d ligand(s), they can be specifically recognized and killed by iNKT cells in an IL-15-dependent process [87]. Other potential iNKT cell TME targets include myeloid-derived suppressor cells (MDSCs). MDSCs have been found to accumulate in the blood, lymph nodes, and bone marrow and at tumor sites in most patients and experimental animals with cancer and inhibit both adaptive and innate immunity [91]. The absence of iNKT cells in mice during influenza virus infection resulted in the expansion of MDSCs, high viral titer, and increased mortality. The adoptive transfer of iNKT cells abolished the suppressive activity of MDSCs and restored virus-specific immune responses, resulting in reduced viral titers and increased rates of host survival [92]. Thus, certain populations of iNKT cells may help alter the TME via their effects on TAMs and MDSCs, to help create a tumor-suppressive or immune-permissive milieu.

### 3.3. Direct Antitumor Cytotoxicity

In addition to their indirect control of tumor growth, iNKT cells can mediate direct killing of tumor targets (see Figure 1(c)). iNKT cells alone, or in combination with NK cells, have been shown to kill a variety of tumor targets* in vitro* [6, 93, 94]. While this mechanism of killing appears to be dependent on the presence of stimulatory glycolipids and CD1d [95, 96], iNKT cell cytotoxicity also appears to be triggered via ligation of NKG2D by target-expressed stress ligands [97]. NKG2D ligation can also costimulate TCR-triggered cytotoxicity [97]. It remains to be seen whether MULT1, the newly identified shed form of high affinity NKG2D ligand that triggers NK-mediated tumor rejection in mice, also activates iNKT cells [98].

Consistent with their direct cytotoxic capacity, iNKT cells express perforin and granzymes, as well as CD178 [34, 96, 99, 100]. In our hands, blockade of CD1d-mediated lipid antigen presentation, disruption of T cell receptor (TCR) signaling, or loss of perforin expression was found to significantly reduce iNKT cell killing* in vitro* [64]. Moreover, we demonstrated that iNKT cells alone were sufficient for control of the growth of a T cell lymphoma* in vivo* that preferentially relies on perforin and the adaptor protein SAP [64, 69]. Mechanistically, iNKT cells rely on SAP for formation of stable conjugates with the tumor targets as well as proper orientation of the lytic machinery at the immunological synapse [69]. Despite the majority of studies implicating iNKT cells as having an antitumor role, a limited number of studies also implicate iNKT cells as suppressing antitumor responses [101], but these paradoxic responses may be related to the level of tumor CD1d expression [102, 103]. Alternatively, these differences may stem from the fact that—contrary to the use of C57BL/6 mice in the previously discussed studies—these last two studies were performed in BALB/c mice, in which there is a predominance of IL-4-producing Th2 phenotype iNKT cells [43].

Interestingly, the antitumor responses of iNKT cells may be regulated by the activity of Type II NKT cells [104]. Terabe et al. demonstrated that Type II variant NKT (vNKT) cells were sufficient for the downregulation of tumor immunosurveillance and relapse growth of a model fibrosarcoma in an antigen-dependent manner [105], while a second study found that activation of vNKT cells with sulfatide antigen could suppress the activation of iNKT cells [106]. These suppressive vNKT cells were found to be predominantly CD4^+^ [107]. Conversely, Type II vNKT cells were, in at least one study, suggested to promote the antitumor activity of CpG oligodeoxynucleotides [108].

iNKT cell antitumor activity is also suppressed by regulatory T (Treg) cells. This suppression appears to be mediated through a contact- and IL-10-dependent mechanism [109, 110]. Indeed, induction of Treg cells suppressed the protective effect of adoptive transfer of iNKT cells into* Jα18−/−* mice [111]. Consistent with these findings, depletion of Treg cells or short-term elimination of their suppressive activity results in enhanced iNKT cell-mediated antitumor responses and increased NK and CD8 T cell activation and IFN-*γ* production [112]. Interestingly, the ability of Treg cells to suppress iNKT cell proliferation depends on the degree of invariant TCR agonism, such that responses to weak (e.g., OCH), but not strong (e.g., *α*-GalCer), agonists were effectively suppressed [110]. When viewed collectively, these findings suggest that iNKT cells possess inherent capacity for direct cytotoxicity but their antigenic exposure may modulate whether their antitumor effects can be suppressed by Treg and vNKT cells.

## 4. iNKT Cell-Based Immunotherapy

Given the preponderance of evidence suggesting that the activation of iNKT cells provides protection against the growth and metastasis of a variety of tumors, safety of *α*-GalCer administration was examined in a Phase I trial [113]. While administration of *α*-GalCer was well tolerated at a range of doses, no clinical responses were observed in patients with advanced solid tumors. On the heels of this study, Nieda et al. showed that treatment of metastatic cancer patients with *α*-GalCer-pulsed immature monocyte-derived DCs resulted in dramatic increases in serum IFN-*γ* and IL-12 and activation of NK and T cells in the majority of subjects. Importantly, this Phase I trial also documented reduction in tumor biomarkers and tumor necrosis in several patients [100]. These findings were extended in a study of a small number of patients, in which the *α*-GalCer-pulsed DCs were matured prior to adoptive transfer. This study demonstrated a >100-fold increase in blood iNKT cell numbers in all patients, and this increase was long-lived (>6 months) [114]. A number of subsequent clinical trials, all with limited number of patients with advanced head and neck or non-small cell lung cancers, have since employed similar strategies of adoptive transfer of *α*-GalCer-pulsed APCs [115–118]. Collectively, these studies demonstrate increases in blood IFN-*γ* levels and iNKT cells in some but not all patients, stabilization of disease in a few of the subjects, and absence of severe treatment-related toxicities.

In a different approach, chemotherapy-refractory 5 lymphoma patients were treated with autologous peripheral blood mononuclear cells (PBMCs) stimulated with anti-CD3, IL-2, and IFN-*γ*. This* ex vivo *stimulation resulted in enrichment of NKT cells (to ~20% on average), and this cell fraction was shown to possess the highest cytotoxic capacity* in vitro*. Of the nine patients who received adoptive transfer of these cells, two showed partial responses and two others had stabilization of disease [119]. Two subsequent studies by Motohashi et al. evaluated the adoptive transfer of* ex vivo *expanded iNKT cell-enriched cells to patients with advanced cancer. In the first, 6 patients with advanced non-small cell lung cancer were treated with either a low or a high dose of* ex vivo *expanded iNKT cells. Of the 3 patients treated with the high dose, all had an increase in the frequency of IFN-*γ*-producing PBMCs and 2 showed expansion of iNKT cells [120]. Although no clinical responses were observed in this study, a follow-up trial of 17 patients with advanced head and neck cancers treated with a high dose of iNKT cell-enriched autologous PBMCs showed a significant increase in IFN-*γ*-producing PBMCs in 10 of 17 patients. Importantly, while none of these patients displayed tumor regression, 5 had disease stabilization and the mean survival time for the subjects with higher frequencies of IFN-*γ*-producing PBMCs was tripled above those with low percentages of IFN-*γ*-producing PBMCs (29.3 versus 9.7 months) [117]. Finally, in a combinational treatment strategy, Kunii et al. administered both* in vitro *expanded iNKT cells and *α*-GalCer-pulsed APCs to patients with advanced head and neck squamous cell carcinomas. Treatment increased the frequencies of iNKT cells and IFN-*γ*-producing PBMCs, and a partial clinical response or disease stabilization was observed in 7 of 8 patients [83]. Although the responses in these studies have not been profound, it must be noted that these iNKT cell-based immunotherapies have all been conducted on patients with advanced malignancies—often those in whom standard chemotherapy, irradiation, and/or surgical excision treatments had failed. Moreover, the majority of these patients had CD1d^−^ cancers.

Future studies of iNKT cell-based immunotherapy may be able to take advantage of two recent technologies. As mentioned previously, many malignancies are associated with a decrease in the numbers and proliferative capacity of peripheral blood iNKT cells. In order to circumvent the difficulty of being able to expand these infrequent and potentially defective cells from patients, Watarai et al. generated induced pluripotent stem (iPS) cells from mature iNKT cells and then expanded large numbers of iNKT cells from these established iPS cells. iPS-NKT cells generated in this fashion were demonstrated to be able to activate and expand antigen-specific CD8 T cell responses to limit the growth of leukemia in mice [121] without inducing graft versus host disease (GvHD) [122]. The second strategy employs chimeric antigen receptors (CARs). Recently, a report by Heczey et al. described iNKT cells engineered to express CARs bearing specificity for GD2, a highly expressed moiety on neuroblastoma cells. In their studies, they showed that iNKT cells expanded from the PBMCs of healthy human donors and transduced with retroviral CAR constructs could protect humanized NSG mice against metastatic neuroblastoma without inducing GvHD [123]. Whether these two technologies could be combined to generate functional CAR-bearing iPS-NKT cells remains to be seen.

## 5. Concluding Remarks

iNKT cells are innate-like effector lymphocytes that not only are directly cytotoxic, but also possess the unique ability to nucleate the antitumor responses of other effector lymphocytes and alter the cellular and angiogenic makeup of the tumor microenvironment. As such, the promise of an effective iNKT cell-based immunotherapy can only be realized by devising and evaluating strategies that simultaneously maximize each of these antitumor effector mechanisms. The challenge for the future will thus be to identify these strategies and apply them to tumors against which iNKT cells wield the most optimal responses.
